# Variation in Hospital Mortality After Complex Cancer Surgery: Patient, Volume, Hospital or Social Determinants?

**DOI:** 10.1245/s10434-023-14852-y

**Published:** 2024-01-09

**Authors:** Muhammad Musaab Munir, Selamawit Woldesenbet, Yutaka Endo, Mary Dillhoff, Jordan Cloyd, Aslam Ejaz, Timothy M. Pawlik

**Affiliations:** Department of Surgery, Wexner Medical Center and James Comprehensive Cancer Center, The Ohio State University, Columbus, OH USA

**Keywords:** Mortality, Variance, Oncologic care, Patient characteristics

## Abstract

**Introduction:**

We sought to define the individual contributions of patient characteristics (PCs), hospital characteristics (HCs), case volume (CV), and social determinants of health (SDoH) on in-hospital mortality (IHM) after complex cancer surgery.

**Methods:**

The California Department of Health Care Access and Information database identified patients who underwent esophagectomy (ES), pneumonectomy (PN), pancreatectomy (PD), or proctectomy (PR) for a malignant diagnosis between 2010 and 2020. Multi-level multivariable regression was performed to assess the proportion of variance explained by PCs, HCs, CV and SDoH on IHM.

**Results:**

A total of 52,838 patients underwent cancer surgery (ES: *n* = 2,700, 5.1%; PN: *n* = 30,822, 58.3%; PD: *n* = 7530, 14.3%; PR: *n* = 11,786, 22.3%) across 294 hospitals. The IHM for the overall cohort was 1.7% and varied from 4.4% for ES to 0.8% for PR. On multivariable regression, PCs contributed the most to the variance in IHM (overall: 32.0%; ES: 21.6%; PN: 28.0%; PD: 20.3%; PR: 39.9%). Among the overall cohort, CV contributed 2.4%, HCs contributed 1.3%, and SDoH contributed 1.2% to the variation in IHM. CV was the second highest contributor to IHM among ES (5.3%), PN (5.3%), and PD (5.9%); however, HCs were a more important contributor among patients who underwent PR (8.0%). The unexplained variance in IHM was highest among ES (72.4%), followed by the PD (67.5%) and PN (64.6%) patient groups.

**Conclusions:**

PCs are the greatest underlying contributor to variations in IHM following cancer surgery. These data highlight the need to focus on optimizing patients and exploring unexplained sources of IHM to improve quality of surgical care.

**Supplementary Information:**

The online version contains supplementary material available at 10.1245/s10434-023-14852-y.

Receipt of high-quality healthcare is hindered by a growing and diverse set of barriers. Potential factors including patient characteristics (PCs; i.e., age, sex, race), hospital characteristics (HCs; i.e., teaching status, nurse-patient ratio), social determinants (i.e., insurance coverage, travel time, social vulnerability index [SVI]) and hospital case volume (CV) have been identified as obstacles to optimal surgical care.^1–5^ In recent years, there has been a significant focus on the centralization of complex cancer operations in order to improve patient cancer outcomes.^6^

The volume-outcome relationship relating to complex surgical procedures has been extensively studied, with high-volume centers demonstrating lower overall morbidity and mortality.^7–10^ Some proponents of regionalization have advocated for the introduction of minimum-volume requirements, which would prohibit hospitals that do not meet the specified threshold from performing high-risk surgical procedures.^11^ Several healthcare systems have committed to implementing such a policy. However, other healthcare networks have been hesitant to embrace a ‘volume pledge’ due to concerns about potential trade-offs between improved outcomes and restricted access to surgical care.^12^ Contemporary studies examining the impact of hospital volume on major surgeries have yielded inconsistent findings relative to postoperative outcomes.^12–15^ A recent systematic review highlighted significant methodological disparities, including variations in how hospitals are categorized, statistical methods employed, and covariates considered that may influence the volume-outcome relationship.^16^ Furthermore, system-wide enhancements in surgical care have reduced surgical mortality over time, attenuating the impact of CV and leading to lower thresholds.^17,18^ Other concerns related to regionalization, such as exacerbation of existing socioeconomic and geospatial disparities, additional travel burden, and fragmentation of surgical care have also been raised.^10^ Hospital volume does not exist in isolation, and there is limited understanding of the broader context and environment in which a person resides. Specifically, the accessibility of essential resources such as food, water, lodging, transportation, employment, and education can vary dramatically according to one’s place of residence.^19^ In turn, these social determinants of health (SDoH) play a significant role in influencing access to high-quality healthcare.^19,20^

As complex cancer surgery becomes more centralized, it is important to examine the volume-outcome relationship through a more holistic lens that considers not only the surrounding SDoH but also individual-level patient factors and hospital structural characteristics. As such, the current study sought to investigate and quantify the role of PCs, HCs, CV, and SDoH on in-hospital mortality (IHM) after high-risk surgical procedures. We hypothesized that hospital volume would not be the most important contributor to the variation in IHM among patients undergoing complex oncologic surgery.

## Methods

### Data Source and Study Population

The California Department of Health Care Access and Information (HCAI) hospital discharge database was utilized to extract data on patients undergoing high-risk oncologic surgery between 2010 and 2020. As a department within the California Health and Human Services Agency, the HCAI is responsible for collecting and distributing healthcare information from licensed healthcare providers and hospitals in California, thereby capturing all hospital stays for patients in the state of California.^10^ Data were de-identified with encrypted ID assignments. Demographic data on the SVI and hospital-level data were obtained from the Centers for Disease Control and Prevention (CDC) SVI and American Hospital Association Survey databases, and subsequently linked using county FIPS codes and the AHAID identifier, respectively.^21,22^ The study was approved and informed consent for de-identified data was waived by the Institutional Review Board of the Ohio State University and the California Committee for the Protection of Human Subjects.

Individuals who underwent a complex oncologic procedure were defined as patients who had an elective resection for esophageal, lung, pancreatic, or rectal malignancies, identified using the International Classification of Diseases, Ninth and Tenth Revision diagnosis and procedure codes (see the Appendix).^4,10^ High-volume hospitals were identified based on established criterion from the Leapfrog Group, which recognizes eight critical procedures with a strong relationship between CV and outcomes.^23^ In particular, the minimum procedural volume thresholds for esophageal, lung, pancreatic, and rectal resections were set at 20, 40, 20, and 16, respectively.^23^

### Primary Exposures and Outcome Interest

Variables included from the California HCAI, AHA and CDC SVI dataset included the following.

1. PCs: Age, sex, race/ethnicity, type of cancer surgery, and Elixhauser comorbidities.
2. HCs: American College of Surgeons (ACS) cancer program accreditation status, teaching status, affiliated medical school, total hospital, and intensive care unit (ICU) beds, total number of operating rooms, and staffing (physician full-time equivalents/bed). Other variables such as equipment available (i.e., interventional radiology, endoscopic retrograde cholangiopancreatography), nurse-patient ratio, trauma facilities and bed size were extracted but were excluded from the analyses due to statistical insignificance.
3. CV: Mean annual hospital CV was computed as a continuous variable to minimize the impact of annual fluctuations in hospital volume.^9^
4. SDoH: Insurance coverage, SVI, and real-world driving time.

The primary outcome of interest was patient IHM, calculated based on disposition at discharge, and the relative contributions of PCs, CV, HCs and SDoH to variations in IHM.

### Geospatial Analysis

Data were processed using ARCGIS Pro (Redlands, CA, USA: Environmental Systems Research Institute, Inc., 2010). Hospitals were geocoded using the reported address in the HCAI database. Network analysis was conducted to evaluate real-world travel distance and time from the centroid of the ZIP code of each patient to the hospital they underwent surgery at using the origin destination (OD) cost matrix function within ARCGIS Pro.^24^ In addition to distance traveled, real-world driving time was utilized as it more accurately reflects the actual impact of travel on patients, particularly in urban and rural areas where driving times may vary greatly.^25^

### Risk- and Reliability-Adjusted Hospital Mortality Groups

To ensure fair comparison among hospitals, IHM rates were reliability-adjusted, ordered based on increasing IHM rates, and subsequently divided into tertiles (low: <33rd percentile; medium: 33rd to 66th percentile; and high: > 66th percentile) based on *a priori* cut-offs to allow for adequate power in the analyses.^26,27^ Reliability adjustment was used to reduce statistical ‘noise’ to allow for more accurate comparisons between facilities reporting risk-adjusted hospital outcomes.^27^ Mortality groups were calculated for each individual operation (i.e., esophagectomy [ES], pneumonectomy [PN], pancreatectomy [PD], and proctectomy [PR]).

### Statistical Analysis

Continuous variables were reported as median with interquartile range (IQR; i.e., PCs) or means with standard deviations (i.e., HCs), and discrete variables were reported as frequencies with percentages. Univariable comparisons were performed using the t-test for continuous variables and Chi-square test for categorical variables. Multi-level mixed-effect logistic regression models were constructed for the overall cohort and each individual procedure to determine the factors associated with IHM, as well as the proportion of variance explained. Four models were constructed that iteratively added (1) PCs; (2) CV; (3) HCs; and (4) SDoH with random intercepts for hospitals to account for clustering to calculate the percentage of variance in IHM explained by the addition of the variables in the model. All models were controlled for age, sex, insurance status, Elixhauser comorbidities, year of diagnosis, cancer type (for overall model), HCs, and social determinants. Sensitivity analyses were performed excluding the ES cohort and revealed no difference in outcomes. Inclusion of specific HCs was determined based on clinical knowledge. Results were reported as odds ratios (ORs) with 95% confidence intervals (CIs). All statistical analyses were derived from two-tailed tests and a *p*-value of <0.05 was considered statistically significant. The analyses were performed using STATA version 17.0 (StataCorp LLC, College Station, TX, USA).

## Results

### Baseline Characteristics of Patients and Hospitals

A total of 52,838 patients underwent a complex oncologic operation (ES: *n* = 2700, 5.1%; PN: *n* = 30,822, 58.3%; PD: *n* = 7530, 14.3%; PR: *n* = 11,786, 22.3%) across 294 hospitals. Overall, median patient age was 68.0 years (60.0–75.0), one-half of patients were male (*n* = 26,526, 50.2%), and most patients were White (*n* = 33,327, 63.1%), and had Medicare (*n* = 30,345, 57.4%) or private (*n* = 16,357, 31.0%) insurance. Smaller subgroups of patients were Black (*n* = 2653, 5.0%), Hispanic (*n* = 7503, 14.2%), Asian (*n* = 7121, 13.5%) or other race/ethnicity (*n* = 2234, 4.2%). Most patients underwent surgery at a high-volume (*n* = 36,037, 68.2%) cancer-accredited center (*n* = 31,571, 59.8%), and traveled a median distance and time of 12.3 miles (5.5–25.8) for 20.0 min (11.8–35.4), respectively, to reach their surgical center of choice. Differences in PCs according to individual cancer procedure are noted in Table 1.

**Table 1:** Baseline characteristics of patients and hospitals

	Overall^a^ [*N* = 52,838]	Esophagectomy^a^ [*n* = 700, 5.1%]	Pneumonectomy^a^ [*n* = 30,822, 58.3%]	Pancreatectomy^a^ [*n* = 7530, 14.3%]	Proctectomy^a^ [*n *= 11,786, 22.3%]	*p*-Value^b^
Patient characteristics						
Age at diagnosis, years	68.0 (60.0–75.0)	66.0 (58.0–72.0)	70.0 (63.0–76.0)	67.0 (60.0–74.0)	61.0 (52.0–70.0)	< 0.001
Sex						< 0.001
Male	26,526 (50.2)	2020 (74.8)	13,614 (44.2)	3847 (51.1)	7045 (59.8)	
Female	26,312 (49.8)	680 (25.2)	17,208 (55.8)	3683 (48.9)	4741 (40.2)	
Race						< 0.001
White	33,327 (63.1)	1689 (62.6)	20,946 (68.0)	4400 (58.4)	6292 (53.4)	
Black	2653 (5.0)	83 (3.1)	1713 (5.6)	413 (5.5)	444 (3.8)	
Hispanic	7503 (14.2)	533 (19.7)	2903 (9.4)	1417 (18.8)	2650 (22.5)	
Asian	7121 (13.5)	264 (9.8)	4161 (13.5)	938 (12.5)	1758 (14.9)	
Other	2234 (4.2)	131 (4.9)	1099 (3.6)	362 (4.8)	642 (5.4)	
Insurance						< 0.001
Medicare	30,345 (57.4)	1355 (50.2)	20,649 (67.0)	4084 (54.2)	4257 (36.1)	
Medicaid	4526 (8.6)	254 (9.4)	1946 (6.3)	634 (8.4)	1692 (14.4)	
Private	16,357 (31.0)	979 (36.3)	7497 (24.3)	2523 (33.5)	5358 (45.5)	
Self-pay	473 (0.9)	33 (1.2)	199 (0.6)	91 (1.2)	150 (1.3)	
Other	1137 (2.2)	79 (2.9)	531 (1.7)	198 (2.6)	329 (2.8)	
Year of diagnosis						< 0.001
2010	4561 (8.6)	155 (5.7)	2996 (9.7)	602 (8.0)	808 (6.9)	
2011	4413 (8.4)	168 (6.2)	2956 (9.6)	602 (8.0)	687 (5.8)	
2012	4306 (8.1)	159 (5.9)	2812 (9.1)	628 (8.3)	707 (6.0)	
2013	4341 (8.2)	187 (6.9)	2794 (9.1)	665 (8.8)	695 (5.9)	
2014	4600 (8.7)	170 (6.3)	2984 (9.7)	700 (9.3)	746 (6.3)	
2015	4109 (7.8)	229 (8.5)	2318 (7.5)	572 (7.6)	990 (8.4)	
2016	5060 (9.6)	312 (11.6)	2662 (8.6)	705 (9.4)	1381 (11.7)	
2017	5400 (10.2)	339 (12.6)	2804 (9.1)	748 (9.9)	1509 (12.8)	
2018	5348 (10.1)	301 (11.1)	2822 (9.2)	734 (9.7)	1491 (12.7)	
2019	5501 (10.4)	346 (12.8)	2951 (9.6)	791 (10.5)	1413 (12.0)	
2020	5199 (9.8)	334 (12.4)	2723 (8.8)	783 (10.4)	1359 (11.5)	
Surgery at high-volume hospital	36,037 (68.2)	570 (21.1)	21,829 (70.8)	5012 (66.6)	8626 (73.2)	< 0.001
Surgery at major teaching hospital	18,571 (35.1)	1373 (50.9)	9614 (31.2)	3892 (51.7)	3692 (31.3)	< 0.001
Surgery at cancer program	31,571 (59.8)	1527 (56.6)	19,205 (62.3)	4151 (55.1)	6688 (56.7)	< 0.001
Miles traveled	12.3 (5.5–25.8)	19.0 (7.6–41.5)	11.3 (5.1–23.7)	16.8 (7.3–37.1)	11.8 (5.4–23.8)	< 0.001
Minutes traveled	20.0 (11.8–35.4)	27.0 (15.0–49.9)	19.0 (11.2–32.8)	25.0 (14.2–47.3)	19.1 (11.5–32.7)	< 0.001
Social Vulnerability Index	59.6 (31.6–77.2)	56.1 (31.6–77.2)	59.6 (31.6–77.2)	56.1 (29.8–77.2)	64.9 (31.6–77.2)	
Elixhauser comorbidities	2.0 (1.0–4.0)	3.0 (2.0–4.0)	3.0 (1.0–4.0)	3.0 (2.0–5.0)	2.0 (1.0–3.0)	
In-hospital mortality	899 (1.7)	120 (4.4)	509 (1.7)	180 (2.4)	90 (0.8)	< 0.001
Hospital characteristics						
Total hospitals	294	158	245	166	255	–
Major teaching hospital	19 (6.5)	18 (11.4)	19 (7.8)	18 (10.8)	17 (6.7)	0.263
Medical school affiliation	151 (51.4)	101 (63.9)	135 (55.1)	106 (36.1)	133 (52.2)	0.030
Cancer program accreditation	108 (36.7)	81 (51.3)	105 (42.9)	82 (49.4)	96 (37.7)	0.022
Physician FTE/bed	0.1 ± 0.36	0.1 ± 0.36	0.1 ± 0.30	0.1 ± 0.43	0.1 ± 0.38	0.948
Mean number of beds	178 ± 199	242 ± 222	202 ± 206	249 ± 216	180 ± 201	0.002
Mean number of operating rooms	8 ± 10	11 ± 12	9 ± 11	11 ± 11	8 ± 10	0.012
Mean annual procedure volume	46.2 ± 69.9	3.7 ± 5.7	28.5 ± 38.6	11.1 ± 20.8	13.5 ± 18.0	<0.001

Statistically significant: *p* < 0.05

^a^Data are expressed as *n* (%), median (IQR), or mean ± standard deviation

^b^Statistical tests performed: Chi-square test of independence, Kruskal-Wallis

*FTE* full-time equivalent, *IQR* interquartile range

Among the 294 hospitals included, 19 (6.5%) were major teaching hospitals, 151 (51.4%) were affiliated with a medical school, and 108 (36.7%) were accredited cancer programs. The mean number of hospital beds per hospital was 178, mean number of operating rooms was 8 per hospital, and the mean physician full-time equivalent (FTE)/bed ratio was 0.1. There were 158 hospitals that performed ES, with a mean annual ES volume of four cases per hospital; 245 hospitals performed PN, with a mean annual PN volume of 29; 166 hospitals performed PD, with a mean annual PD volume of 11 cases per hospital; and 255 hospitals performed PR with a mean annual PR volume of 14 cases per hospital.

The IHM for the overall cohort was 1.7% (*n* = 899) and decreased significantly from 2.2% in 2010 to 1.2% in 2020 (*p* < 0.001). IHM varied according to cancer procedure, ranging from 4.4% for ES, 2.4% for PD, 1.7% for PN, and 0.8% for PR (*p* < 0.001). Patients who experienced IHM were more likely to be older (73.0 years vs. 68.0 years), have a greater median Elixhauser comorbidity index (5.0 vs. 2.0), were more frequently male (64.4% vs. 50.0%), had Medicare insurance (74.6% vs. 57.1%), and resided in areas with higher SVI (71.9 vs. 56.1) [all *p* < 0.001]. In contrast, patients treated at high-volume (68.6% vs. 47.4%), cancer-accredited (59.8% vs. 56.0%) or teaching (35.3% vs. 24.4%) hospitals, as well as patients with private insurance (31.2% vs. 14.2%) and who traveled longer distances (12.4 vs. 9.4 miles) and had longer travel times (20.1 vs. 16.9 min) were less likely to experience an IHM event (all *p* < 0.001) [Table 2].

**Table 2:** Patient characteristics and in-hospital mortality

Patient characteristics	Alive [*n* = 51,939]	In-hospital mortality [*n* = 899, 5.1%]	*p*-Value^b^
Age at diagnosis, years	68.0 (60.0–75.0)	73.0 (66.0–79.0)	< 0.001
Sex			< 0.001
Male	25,947 (50.0)	579 (64.4)	
Female	25,992 (50.0)	320 (35.6)	
Race			0.125
White	32,769 (63.1)	558 (62.1)	
Black	2601 (5.0)	52 (5.8)	
Hispanic	7357 (14.2)	146 (16.2)	
Asian	7019 (13.5)	102 (11.3)	
Other	2193 (4.2)	41 (4.6)	
Insurance			< 0.001
Medicare	29,674 (57.1)	671 (74.6)	
Medicaid	4449 (8.6)	77 (8.6)	
Private	16,229 (31.2)	128 (14.2)	
Self-pay	467 (0.9)	6 (0.7)	
Other	1120 (2.2%)	17 (1.9)	
Surgery at high-volume hospital	35,611 (68.6%)	426 (47.4)	< 0.001
Surgery at major teaching hospital	18,352 (35.3%)	219 (24.4)	< 0.001
Surgery at cancer program	31,068 (59.8%)	503 (56.0)	< 0.001
Miles traveled	12.4 (5.5–25.9)	9.4 (4.5–20.9)	< 0.001
Minutes traveled	20.1 (11.8–35.5)	16.9 (10.2–29.1)	< 0.001
Social Vulnerability Index	56.1 (31.6–77.2)	71.9 (33.3–77.2)	
Elixhauser comorbidities	2.0 (1.0–4.0)	5.0 (4.0–7.0)	< 0.001

Statistically significant: *p* < 0.05

^a^Data are expressed as *n* (%) or median (IQR)

^b^Statistical tests performed: Chi-square test of independence, Kruskal–Wallis

*IQR* interquartile range

### Patient and Hospital Characteristics Across Reliability-Adjusted Mortality Groups

In assessing individual PCs across low, medium, and high mortality groups, patients from the ES subgroup were more likely to undergo surgery at a low-mortality hospital versus a high- or medium-mortality center (5.9% vs. 4.5% and 4.9%). Patients from the high-mortality group were more likely to be covered with Medicaid (9.9% vs. 9.2% and 6.6%), have a higher median Elixhauser comorbidity index (3.0 vs. 2.0 for both), and be of Hispanic (15.0% vs. 14.2% and 13.4%) or Asian (14.3% vs. 12.5% and 13.6%) race/ethnicity compared with the medium- and low-mortality groups. In contrast, patients in the low-mortality group were more likely to go to a high-volume (88.8% vs. 58.1% and 57.1%), cancer-accredited (64.6% vs. 62.1% and 52.4%) or teaching (50.5% vs. 34.1% and 20.5%) hospital, have private insurance (34.2% vs. 29.6% and 29.0%), and have greater median travel distances (15.1 vs. 11.1 and 11.3 miles) and times (24.1 vs. 18.6 and 17.9 min) versus patients with medium and high mortality (Table 1 in the electronic supplementary material [ESM]).

**Fig. 1:**
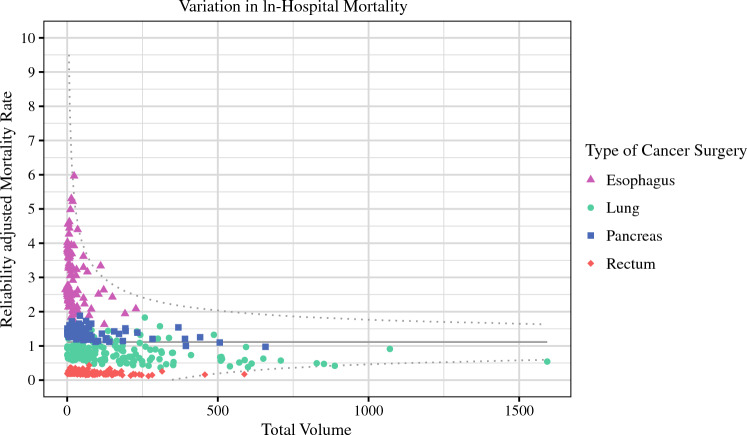
Funnel plot presenting variation in reliability-adjusted hospital mortality rates across different types of complex cancer surgery

The variability in IHM across reliability-adjusted mortality rates relative to hospital CV is presented in Fig. 1. After classification into mortality groups, most hospitals fell into the high-mortality group for the ES (*n* = 90, 60.0%), PD (*n* = 92, 55.4%) and PR (*n* = 134, 52.5%) cohorts; however, most of the hospitals among the PN subgroup were more likely to belong to the medium mortality group (*n* = 146, 59.6%) [all *p* < 0.001]. Notably, hospitals in the lowest mortality group had the highest mean annual CV (ES: 11.1 ± 12.2; PN: 76.6 ± 66.6; PD: 45.2 ± 41.7; PR: 36.7 ± 25.7), mean number of hospital beds (ES: 380 ± 236; PN: 376 ± 236; PD: 292 ± 187; PR: 369 ± 246), and mean number of operating rooms (ES: 21 ± 14; PN: 18 ± 15; PD: 17 ± 12; PR: 21 ± 16) [all *p* < 0.001]. The variation in facility characteristics across hospital mortality groups is noted in ESM Table 2.

### Multivariable Analysis and Proportion of Variance Explained

On multivariable logistic regression analysis, PCs, hospital volume, and social determinants were significantly associated with IHM. Notably, increasing age (1-year increase; OR 1.03, 95% CI 1.02–1.04), Hispanic (reference: White; OR 1.36, 95% CI 1.04–1.76) and Asian (reference: White; OR 1.35, 95% CI 1.00–1.82) race/ethnicity, greater Elixhauser comorbidity index (1 unit increase; OR 1.63, 95% CI 1.58–1.68), SVI (1 unit increase; OR 1.01, 95% CI 1.01–1.01), as well as Medicare (reference: private; OR 1.36, 95% CI 1.04–1.78) and Medicaid (reference: private; OR 2.00, 95% CI 1.31–3.04) insurance were associated with higher IHM (all *p* < 0.05). In contrast, female sex (reference: male; OR 0.66, 95% CI 0.56–0.78), cancer surgery type (reference: ES; PN: OR 0.31, 95% CI 0.23–0.44; PD: OR 0.45, 95% CI 0.33–0.61; PR: OR 0.22, 95% CI 0.15–0.32), higher mean hospital volume (10-procedure increase; OR 0.99, 95% CI 0.98–1.00), and further driving times (10 min increase; OR 0.95, 95% CI 0.93–0.98) were associated with lower IHM (all *p* < 0.05).

**Fig. 2:**
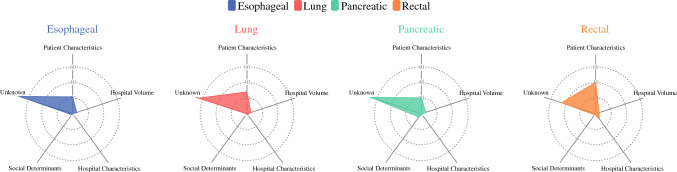
Spider chart demonstrating the distribution of variance explained by patient characteristics, hospital case volume, hospital characteristics, social determinants of health, and unknown factors across different types of complex cancer surgery

The distribution of variance explained by PCs, CV, HCs, SDoH, and unknown factors is illustrated in Fig. 2. PCs contributed the most to the variance in IHM among the overall cohort (32.0%), as well as each individual operation (ES: 21.6%; PN: 28.0%; PD: 20.3%; PR: 39.9%). Of note, CV contributed just 2.4%, HCs contributed 1.3%, and SDoH contributed 1.2% to the variation in IHM among the overall cohort. Interestingly, CV was the second highest contributor to IHM among ES (5.3%), PN (5.3%), and PD (5.9%); however, HCs were a more important contributor among patients who underwent PR (8.0%). SDoH contributed <2.0% to the variation in IHM for all cancer surgery subtypes except for PD (5.1%). The unexplained variance in IHM was highest among ES (72.4%) followed by the PD (67.5%), PN (64.6%), and PR (45.5%) subgroups, respectively (Table 3).

**Table 3:** Multi-level, mixed-effect logistic regression analysis for in-hospital mortality

Variable	Comparison	Model 1		Model 2		Model 3		Model 4	
		OR 95% CI	*p*-Value	OR 95% CI	*p*-Value	OR 95% CI	*p*-Value	OR 95% CI	*p*-Value
*Patient characteristics*									
Age	Per year increase	1.03 1.02–1.04	< 0.001	1.03 1.02–1.04	<0.001	1.03 1.02–1.04	<0.001	1.03 1.02–1.04	<0.001
Sex	Female vs. male	0.63 0.55–0.73	< 0.001	0.63 0.55–0.72	<0.001	0.66 0.55–0.78	<0.001	0.66 0.56–0.78	<0.001
Race	Black vs. White	1.12 0.84–1.49	0.885	1.10 0.82–1.46	0.521	0.99 0.71–1.38	0.957	0.87 0.63–1.20	0.398
	Hispanic vs. White	1.47 1.18–1.83	0.001	1.38 1.11–1.71	0.004	1.50 1.18–1.91	0.001	1.36 1.04–1.76	0.023
	Asian vs. White	1.26 1.00–1.60	0.054	1.26 1.00–1.58	0.047	1.38 1.03–1.84	0.047	1.35 1.00–1.82	0.047
	Other vs. White	1.44 0.97–2.15	0.073	1.51 1.01–2.25	0.045	1.67 1.04–2.68	0.045	1.56 0.95–2.55	0.076
Type of Cancer	Lung vs. esophageal	0.37 0.28–0.48	< 0.001	0.32 0.25–0.42	<0.001	0.35 0.25–0.48	<0.001	0.31 0.23–0.44	<0.001
	Pancreatic vs. esophageal	0.46 0.35–0.59	< 0.001	0.47 0.36–0.61	<0.001	0.49 0.36–0.65	<0.001	0.45 0.33–0.61	<0.001
	Rectal vs. esophageal	0.27 0.19–0.37	< 0.001	0.23 0.17–0.32	<0.001	0.23 0.11–0.34	<0.001	0.22 0.15–0.32	<0.001
Elixhauser comorbidities	Per 1-unit increase	1.63 1.59–1.68	< 0.001	1.62 1.57–1.65	<0.001	1.64 1.59–1.70	<0.001	1.63 1.58–1.68	<0.001
*Hospital volume*									
Mean procedural volume	Per 10-procedure increase			0.98 0.97–0.99	<0.001	0.98 0.97–0.99	0.005	0.99 0.98–1.00	0.015
*Hospital characteristics*									
Major teaching hospital	Teaching vs non-teaching					1.23 0.89–1.70	0.220	1.04 0.76–1.41	0.814
Medical school affiliation	Affiliation vs no affiliation					0.76 0.57–1.00	0.050	0.86 0.66–1.12	0.264
Cancer program accreditation	Accreditation vs non-accreditation					0.82 0.64–1.06	0.126	0.88 0.69–1.11	0.272
Physician FTE-bed ratio	Per 1 FTE increase per bed					0.89 0.78–1.02	0.100	0.96 0.85–1.07	0.433
*Social determinants*									
Social Vulnerability Index	Per 1 unit increase							1.01 1.01–1.01	<0.001
Insurance	Medicare vs. private							1.36 1.04–1.78	0.023
	Medicaid vs. private							2.00 1.31–3.04	0.001
	Self-pay vs. private							1.57 0.42–5.92	0.506
	Other vs. private							1.89 1.00–3.56	0.050
Time to destination hospital	Per 10 min increase							0.95 0.93–0.98	0.001
Proportion of variance explained		32.0%		34.3%		35.6%		36.8%	

Bolded data indicate statistical significance: *p* < 0.05

*OR* odds ratio, *CI* confidence interval, *FTE* full-time equivalents

All multivariable regression models were adjusted for age, sex, race, insurance status, all 27 Elixhauser comorbidities, year of diagnosis, cancer type, hospital characteristics and clustered at the hospital-level

## Discussion

The provision of high-quality surgical services for cancer patients is crucial to ensure equitable healthcare. Rapid advancements in healthcare infrastructure and technology have ensured that regionalization of complex oncologic surgical care into large, multidisciplinary centers is likely to persist. However, recent studies examining the volume-outcome relationship have yielded conflicting results and raised several concerns regarding substantial methodological disparities, reduction in surgical mortality over time that have led to attenuation of the impact of CV, and worsening inequities in access to care for vulnerable populations.^6,8,9,16–18^ Moreover, the role of SDoH relative to surgical outcomes has been increasingly recognized over the last decade.^19,28,29^ For instance, recent work from our own group has emphasized the importance of neighborhood characteristics and social vulnerability on operative outcomes.^30,31^ The effect of hospital volume on postoperative outcomes does not exist in a vacuum, and the impact of other patient, hospital, and neighborhood-level factors on this association remains relatively ill-defined. Therefore, the current work was important as we specifically quantified the role of PCs, HCs, CV, and SDoH on IHM after complex oncologic surgery. Of note, the current study demonstrated that PCs contribute most to IHM (ranging from 20.3% in pancreatic surgery to 39.9% in rectal surgery) after major cancer surgery in the state of California. CV accounted for 4.9–5.9%, HCs between 1.0 and 8.0%, and SDoH was responsible for 0.4–5.1% of the variability in IHM. Importantly, more than one-half of the variance in IHM after esophageal, lung and pancreatic surgery remained unexplained.

Despite advancements in the delivery of cancer care at the national level, there is still significant variation in patient health outcomes. The current study builds on previous work from Martins et al. that examined variations in hospital mortality rates among a 2006–2011 surgical cohort, by exploring the role of additional contributors such as social determinants alongside patient- and hospital-level characteristics among a high-risk cancer surgery cohort during a more recent time period (2010–2020).^26^ Similar to the previous study, we noted that patient-level factors were the single most important contributor to IHM across hospitals in California, thereby highlighting the importance of risk identification, stratification and mitigation to improve postoperative outcomes. While the urgency associated with many surgical procedures often limits the potential for substantial improvements in patient functional status and health before surgery, the rapid rise in utilization of neoadjuvant therapy among cancer patients may provide a window of opportunity. Even short-term behavioral modifications such as quitting smoking have been associated with improved healthcare outcomes. Other noteworthy approaches include patient prehabilitation, which involves the focused optimization of modifiable risk factors, both physical and psychological, prior to undergoing surgery.^32^ Prehabilitation has lead to better postoperative outcomes, including reduced morbidity, lower IHM, shorter hospital stays, enhanced patient quality of life, faster recovery times, and decreased need for additional interventions.^32^ Nonetheless, implementing prehabilitation on a larger scale poses challenges and requires coordination among patients, social services, stakeholders and healthcare organizations. The Strong for Surgery program, initially launched at the University of Washington in 2012 and subsequently integrated as an ACS Quality Program may serve as an archetype of a successful prehabilitation effort.^33^ Today, this program has been adopted by more than 200 hospitals in the United States and engages various stakeholders within the healthcare system, including patients themselves, to optimize patient readiness before surgery.^33^ Moreover, prehabilitation also plays a significant role in the Enhanced Recovery after Surgery (ERAS) programs, which have gained momentum in US hospitals over the last two decades and have consistently been associated with improved surgical outcomes.^34–36^

The current study also examined the impact of structural characteristics within hospitals on variation in IHM, particularly in the context of surgery for rectal cancer (8.0%). Hospitals with advanced clinical services are more capable in preventing the incidence of postoperative complications, as well as in managing them effectively, thereby reducing the ‘failure to rescue’ phenomenon that is associated with high IHM rates.^37–39^ HCs such as availability of equipment and advanced medical technology, the capacity and care models within ICUs, staffing models, teaching status, and easy accessibility to essential healthcare professionals such as intensivists, rapid response teams, nurse practitioners, and physician assistants play a pivotal role in preventing the occurrence of failure to rescue, as these factors help to identify hospitals that are better equipped to recognize and effectively manage severe postoperative complications.^40,41^ For instance, recent research has demonstrated that the presence of a dedicated cancer program within a hospital is a more reliable predictor of IHM than surgical volume or other hospital attributes.^42^ Although certain fundamental structural characteristics of hospitals may be deemed essential for performing high-risk operations, implementing and sustaining such changes at the individual hospital level can present considerable difficulties. Consequently, interim strategies, such as directing higher-risk patients to hospitals with greater resources, may be explored to enhance clinical outcomes.^43^

Another important consideration that has gained traction over recent years is the role of SDoH. Although it may be useful to compare patients based on factors such as education, transportation, and financial resources, these assessments do not provide a comprehensive understanding of the holistic ‘lived experience’ of individuals within a particular community. Healthcare research and policy efforts that consider this wider social and environmental context might prove more effective, as social determinants and non-medical social needs can exert a significant impact on an individual’s overall well-being.^19,20,28^ To this effect, social vulnerability functions as a measure of the resilience of a community to external stressors and has been identified as a possible root cause of health inequities.^30,31^ Moreover, patient travel patterns function as another key determinant that impact access to high-quality surgical care.^10,44,45^ Patients may seek treatment at designated cancer centers in the hope of realizing improved outcomes; however, delays in appropriate cancer treatment to reach such centers can be problematic and can even result in worse cancer outcomes.^45–49^ It is also imperative to acknowledge the role of patient preferences, a factor that is often underestimated by policymakers and stakeholders. A seminal study conducted by Finlayson et al. revealed that 45% of patients preferred to receive treatment at a local medical center, even when the risk of mortality following surgery was twice as high there versus a regional center with half the mortality risk that was further away.^50^ Similarly, most patients undergoing gastrectomy in the state of California chose to forgo evidence-based decision making in order to undergo surgical treatment at centers closer to their area of residence.^51^ Yet another study highlighted that 74% of patients faced considerable financial and insurance-related barriers to long-distance travel for healthcare.^52^ Nevertheless, most of these patients indicated a willingness to travel further if some of these barriers could be mitigated.^52^ The current work is in line with these studies and demonstrates a reduction in IHM among patients who traveled further and longer to undergo high-risk surgical care. Collectively, these studies underscore the importance of addressing barriers to ensure equitable access when implementing policies based on the volume-outcome association.

Results from the current work also raise doubts on the appropriateness of utilizing arbitrary hospital CV thresholds, as proposed by initiatives such as the Volume Pledge, to drive health quality improvement. If contemporary hospital volume criteria are applied, over 70% of hospitals would be disqualified from performing complex oncologic procedures such as esophageal, lung, pancreatic or rectal surgery.^17,53^ Additionally, strict implementation of volume thresholds to promote regionalization of high-risk surgical care may further exacerbate inequities in healthcare access and contribute to the fragmentation of care, particularly among disadvantaged populations.^4,10,54^ Notwithstanding the fact that hospital CV is aimed at improving healthcare outcomes by increasing the number of cases as a quantifiable quality improvement measure, the past two decades and significant advancements in surgical research have demonstrated that this approach fails to consider factors such as patient preferences, geospatial access barriers, and patterns of insurance referrals. While there is undeniable evidence linking CV to outcomes, too much attention has been placed on the actual number of cases, thereby neglecting a more holistic approach to identifying areas for improvement. A potentially more effective and equitable approach would concentrate on patient prehabilitation, improvement of hospital resources, and engagement of socioeconomically vulnerable communities. Moreover, it is crucial that unexplained sources of variance in IHM are promptly identified. These unknown sources are likely related to complex factors that are difficult to capture in healthcare databases, such as the implementation of continuous quality improvement (CQI) initiatives, a surgical center’s culture of safety, and the multifaceted interactions between patients’ comorbidities, surgical procedures, and postoperative outcomes. Investigating these underlying factors could be facilitated by analyzing data with more clinical granularity, utilizing advanced artificial language methodologies such as natural language processing and machine learning, and employing robust statistical models such as mediation analyses.^55,56^

There are several limitations to consider when interpreting the current study results. Although, the California database provided for 100% capture and comprehensive data gathering on travel for all patients having surgery at California-licensed facilities, the data were restricted to one state. As a result, these findings may not be generalizable to other states with different geographies. Travel distance and time were calculated using StreetMap Premium in ARCGIS Pro geographical software, and while this was more accurate than straight-line travel distance studies, it may not have fully reflected real-world traffic patterns as certain datapoints on time of day traveled could not be assessed. Moreover, patients who rely on public transportation, especially those with limited resources, may experience significantly longer travel times due to the availability and efficiency of such services. County-level data were used for the calculation of SVI, hence there might have been residual heterogeneity. Larger counties, for instance, might have had inhabitants with a wide range of socioeconomic vulnerability. The primary endpoint of the study was IHM, thereby restricting the findings from extrapolation to alternative outcomes such as 30- or 90-day mortality that may have different proportionate contributions to variations in outcomes. Due to the restrictions of the HCAI database, the current study was unable to examine disease stage or long-term outcomes such as disease-free survival. Furthermore, admissions for selected operations were assessed rather than diagnosis of incident malignancy. While a distinction between locally advanced, regional, or metastatic disease could not be made, most patients with metastatic illness would not have been given the option of a resection, particularly for pancreatic and esophageal cancer. Finally, some individuals with potentially operable cancer might not have undergone surgery and as such were left out of the study cohort.

## Conclusion

The current study demonstrates that most of the variability in IHM following complex cancer surgery stems from patient-level factors rather than hospital CV, HCs or social determinants. Furthermore, over half the variation in IHM remains unexplained, especially among esophageal, lung and pancreatic cancer. These data highlight the need to focus on patient optimization and on risk reduction in addition to initiatives that are aimed at regionalization, structural quality improvement and social determinants. Moreover, there is a need to explore the reasons for the hitherto unexplained factors that underlie IHM to improve the quality of surgical care.

### Supplementary Information

Below is the link to the electronic supplementary material.

Supplementary file1 (DOCX 22 kb)

## Data Availability

The data for this study were obtained from the California Office of State-Wide Health Planning and Development (OSHPD) hospital discharge database. There are restrictions to the availability of this data, which is used under license for this study. Data can be accessed with permission from the California Department of Health Care Access and Information.
