# Supplementary material for: Variation in Hospital Mortality After Complex Cancer Surgery: Patient, Volume, Hospital or Social Determinants?
Source: Ann Surg Oncol. 2024 Jan 9;31(5):2856–66. doi: 10.1245/s10434-023-14852-y (PMC10997543; doi:10.1245/s10434-023-14852-y)
Supplement: Supplementary file 1 — Supplementary file1 (DOCX 22 kb) [file 10434_2023_14852_MOESM1_ESM.docx]

**Supplementary Table 1.** Baseline characteristics of patients across reliability-adjusted in-hospital mortality groups.

| **Patient Characteristics** | **Reliability-adjusted In-hospital Mortality Group** | | | **p-value*** |
| --- | --- | --- | --- | --- |
|  | **Low** | **Medium** | **High** |  |
| Age at diagnosis (years) | 68.0 (59.0-74.0) | 68.0 (60.0-75.0) | 68.0 (60.0-75.0) | <0.001 |
| Sex |  |  |  | <0.001 |
| Male | 9,020 (50.2%) | 8,692 (49.9%) | 8,814 (50.5%) |  |
| Female | 8,938 (49.8%) | 8,736 (50.1%) | 8,638 (49.5%) |  |
| Race |  |  |  | <0.001 |
| White | 11,581 (64.5%) | 11,008 (63.2%) | 10,738 (61.5%) |  |
| Black | 761 (4.2%) | 1,012 (5.8%) | 880 (5.0%) |  |
| Hispanic | 2,399 (13.4%) | 2,480 (14.2%) | 2,624 (15.0%) |  |
| Asian | 2,439 (13.6%) | 2,186 (12.5%) | 2,496 (14.3%) |  |
| Other | 778 (4.3%) | 742 (4.3%) | 714 (4.1%) |  |
| Insurance |  |  |  | <0.001 |
| Medicare | 10,156 (56.6%) | 10,111 (58.0%) | 10,078 (57.7%) |  |
| Medicaid | 1,180 (6.6%) | 1,612 (9.2%) | 1,734 (9.9%) |  |
| Private | 6,142 (34.2%) | 5,159 (29.6%) | 5,056 (29.0%) |  |
| Self-pay | 172 (1.0%) | 169 (1.0%) | 132 (0.8%) |  |
| Other | 308 (1.7%) | 377 (2.2%) | 452 (2.6%) |  |
| Year of diagnosis |  |  |  | <0.001 |
| 2010 | 1,597 (8.9%) | 1,512 (8.7%) | 1,452 (8.3%) |  |
| 2011 | 1,522 (8.5%) | 1,457 (8.4%) | 1,434 (8.2%) |  |
| 2012 | 1,401 (7.8%) | 1,395 (8.0%) | 1,510 (8.7%) |  |
| 2013 | 1,516 (8.4%) | 1,358 (7.8%) | 1,467 (8.4%) |  |
| 2014 | 1,554 (8.7%) | 1,467 (8.4%) | 1,579 (9.0%) |  |
| 2015 | 1,412 (7.9%) | 1,237 (7.1%) | 1,460 (8.4%) |  |
| 2016 | 1,668 (9.3%) | 1,658 (9.5%) | 1,734 (9.9%) |  |
| 2017 | 1,806 (10.1%) | 1,820 (10.4%) | 1,774 (10.2%) |  |
| 2018 | 1,834 (10.2%) | 1,756 (10.1%) | 1,758 (10.1%) |  |
| 2019 | 1,869 (10.4%) | 1,950 (11.2%) | 1,682 (9.6%) |  |
| 2020 | 1,779 (9.9%) | 1,818 (10.4%) | 1,602 (9.2%) |  |
| Cancer procedure type |  |  |  | <0.001 |
| Esophagectomy | 1,061 (5.9%) | 858 (4.9%) | 781 (4.5%) |  |
| Pneumonectomy | 10,388 (57.8%) | 10,191 (58.5%) | 10,243 (58.7%) |  |
| Pancreatectomy | 2,515 (14.0%) | 2,506 (14.4%) | 2,509 (14.4%) |  |
| Proctectomy | 3,994 (22.2%) | 3,873 (22.2%) | 3,919 (22.5%) |  |
| Surgery at high-volume hospital | 15,946 (88.8%) | 10,119 (58.1%) | 9,972 (57.1%) | <0.001 |
| Surgery at major teaching hospital | 9,062 (50.5%) | 5,935 (34.1%) | 3,574 (20.5%) | <0.001 |
| Surgery at cancer program | 11,607 (64.6%) | 10,818 (62.1%) | 9,146 (52.4%) | <0.001 |
| Miles traveled | 15.1 (6.5-32.3) | 11.1 (5.1-23.2) | 11.3 (5.1-23.4) | <0.001 |
| Minutes traveled | 24.1 (13.6-43.4) | 18.6 (11.2-32.2) | 17.9 (10.9-30.6) | <0.001 |
| Social Vulnerability Index | 56.1 (29.8-77.2) | 64.9 (31.6-77.2) | 47.4 (31.6-77.2) | <0.001 |
| Elixhauser comorbidities | 2.0 (1.0-4.0) | 2.0 (1.0-4.0) | 3.0 (1.0-4.0) | <0.001 |

*^Note^*^: statistically significant: p<0.05
 a Statistics presented: n (%), median (IQR), mean ± standard deviation.
 * Statistical tests performed:^ *^χ^*^2 test of independence, Kruskal-Wallis.^

**Supplementary Table 2.** Hospital characteristics across reliability-adjusted in-hospital mortality groups.

| **Hospital Characteristics** | **Reliability-adjusted In-hospital Mortality Group** | | | **p-value*** |
| --- | --- | --- | --- | --- |
|  | **Low** | **Medium** | **High** |  |
| **Esophagectomy** |  |  |  |  |
| Total hospitals | 17 | 51 | 90 | – |
| Major teaching hospital | 5 (29.4%) | 7 (13.7%) | 6 (6.7%) | 0.021 |
| Medical school affiliation | 2 (11.8%) | 19 (37.3%) | 36 (40.0%) | 0.083 |
| Cancer program accreditation | 10 (58.8%) | 28 (54.9%) | 43 (47.8%) | 0.578 |
| Physician FTE/bed | 0.0 ± 0.0 | 0.1 ± 0.5 | 0.0 ± 0.2 | 0.639 |
| Mean number of beds | 380 ± 236 | 269 ± 210 | 201 ± 215 | 0.005 |
| Mean number of operating rooms | 21 ± 14 | 12 ± 12 | 8 ± 10 | <0.001 |
| Mean annual procedure volume | 11.1 ± 12.2 | 3.7 ± 4.7 | 2.3 ± 2.2 | <0.001 |
| **Pneumonectomy** |  |  |  |  |
| Total hospitals | 26 | 146 | 73 | – |
| Major teaching hospital | 6 (23.1%) | 8 (5.5%) | 5 (6.8%) | 0.008 |
| Medical school affiliation | 21 (80.8%) | 74 (50.7%) | 40 (54.8%) | 0.018 |
| Cancer program accreditation | 18 (69.2%) | 53 (36.3%) | 34 (46.6%) | 0.006 |
| Physician FTE/bed | 0.2 ± 0.7 | 0.0 ± 0.2 | 0.0 ± 0.0 | 0.078 |
| Mean number of beds | 376 ± 236 | 167 ± 177 | 209 ± 218 | <0.001 |
| Mean number of operating rooms | 18 ± 15 | 7 ± 8 | 9 ± 11 | <0.001 |
| Mean annual procedure volume | 76.6 ± 66.6 | 19.4 ± 26.1 | 29.3 ± 33.4 | <0.001 |
| **Pancreatectomy** |  |  |  |  |
| Total hospitals | 13 | 61 | 92 | – |
| Major teaching hospital | 5 (38.5%) | 8 (13.1%) | 5 (5.4%) | 0.001 |
| Medical school affiliation | 13 (100.0%) | 44 (72.1%) | 49 (53.3%) | 0.001 |
| Cancer program accreditation | 8 (61.5%) | 33 (54.1%) | 41 (44.6%) | 0.339 |
| Physician FTE/bed | 0.0 ± 0.0 | 0.2 ± 0.6 | 0.0 ± 0.2 | 0.284 |
| Mean number of beds | 292 ± 187 | 283 ± 226 | 220 ± 210 | 0.160 |
| Mean number of operating rooms | 17 ± 12 | 13 ± 13 | 9 ± 9 | 0.012 |
| Mean annual procedure volume | 45.2 ± 41.7 | 10.1 ± 18.0 | 7.0 ± 12.6 | <0.001 |
| **Proctectomy** |  |  |  |  |
| Total hospitals | 28 | 93 | 134 | – |
| Major teaching hospital | 7 (25.0%) | 5 (5.4%) | 5 (3.7%) | <0.001 |
| Medical school affiliation | 24 (85.7%) | 46 (49.5%) | 63 (47.0%) | <0.001 |
| Cancer program accreditation | 17 (60.7%) | 30 (32.3%) | 49 (36.6%) | 0.023 |
| Physician FTE/bed | 0.2 ± 0.7 | 0.1 ± 0.5 | 0.0 ± 0.1 | 0.157 |
| Mean number of beds | 369 ± 246 | 165 ± 180 | 151 ± 185 | <0.001 |
| Mean number of operating rooms | 21 ± 16 | 6 ± 8 | 6 ± 8 | <0.001 |
| Mean annual procedure volume | 36.7 ± 25.7 | 11.9 ± 15.8 | 9.7 ± 13.5 | <0.001 |

*^Note^*^: statistically significant: p<0.05
a Statistics presented: n (%), median (IQR), mean ± standard deviation.
* Statistical tests performed:^ *^χ^*^2 test of independence, Kruskal-Wallis.^
